# Searching for Stable Si_n_C_n_ Clusters: Combination of Stochastic Potential Surface Search and Pseudopotential Plane-Wave Car-Parinello Simulated Annealing Simulations

**DOI:** 10.3390/molecules18078591

**Published:** 2013-07-22

**Authors:** Xiaofeng F. Duan, Larry W. Burggraf, Lingyu Huang

**Affiliations:** 1 Air Force Research Laboratory DoD Supercomputer Resource Center, Wright-Patterson Air Force Base, OH 45433, USA; 2 Air Force Institute of Technology, Wright-Patterson Air Force Base, OH 45433, USA; 3 College of Agriculture, Food Science and Sustainable Systems, Kentucky State University, Frankfort, KY 40601, USA

**Keywords:** Si_n_C_n_ cluster, stochastic potential surface search, simulated annealing simulation, DFT optimization, carbon/silicon segregation

## Abstract

To find low energy Si_n_C_n_ structures out of hundreds to thousands of isomers we have developed a general method to search for stable isomeric structures that combines Stochastic Potential Surface Search and Pseudopotential Plane-Wave Density Functional Theory Car-Parinello Molecular Dynamics simulated annealing (PSPW-CPMD-SA). We enhanced the Sunders stochastic search method to generate random cluster structures used as seed structures for PSPW-CPMD-SA simulations. This method ensures that each SA simulation samples a different potential surface region to find the regional minimum structure. By iterations of this automated, parallel process on a high performance computer we located hundreds to more than a thousand stable isomers for each Si_n_C_n_ cluster. Among these, five to 10 of the lowest energy isomers were further optimized using B3LYP/cc-pVTZ method. We applied this method to Si_n_C_n_ (**n** = 4–12) clusters and found the lowest energy structures, most not previously reported. By analyzing the bonding patterns of low energy structures of each Si_n_C_n_ cluster, we observed that carbon segregations tend to form condensed conjugated rings while Si connects to unsaturated bonds at the periphery of the carbon segregation as single atoms or clusters when **n** is small and when **n** is large a silicon network spans over the carbon segregation region.

## 1. Introduction

Interest is growing in understanding the structures of molecular clusters intermediate between small molecules and nano-crystals of carbon, silicon and silicon carbide. Study of the complex molecular clusters of silicon carbide builds on better studied understanding of carbon and silicon molecular clusters. As is the case for thin-film silicon and carbon, an important interest for silicon carbide is for applications of solid state thin film materials. Single crystal silicon carbide is important for sensors and electronics and nano-mechanics applications, especially for these applications in challenging environments.

Formation of high-purity, high-performance thin film materials from small molecules is a general approach for generating pure thin film materials for electronics and nano-materials applications using processing techniques such as chemical vapor deposition, molecular beam epitaxy or deposition by pulsed laser ablation [1,2]. Structures of solid state carbon and silicon are very different from structures of small carbon and silicon molecules and still different from structures of carbon and silicon molecular clusters. Interesting bonded structures confer special stability or reactivity. For different synthesis processes and in various processing conditions the growth of stable intermediate molecular clusters can either benefit or can degrade efficient growth of single crystal thin films from small molecules. In addition to interest in applications of thin film materials new applications of nano-crystals also motivates this study.

More recent applications of nanometer-scale particulate materials take advantage of the exceptional optical [3] and photochemical [4,5] and biological properties [6] of materials having sizes intermediate between small molecules and solid state materials. Study of molecular clusters as examples and models of practical nano-particles can provide insight into the stability and reactivity and optical properties leading to their special characteristics.

Here again, study of silicon nano-particles and carbon allotropes, buckyballs and graphene, point toward potential for discovery of novel properties of silicon carbide nano-particles. For example, silicon nanocrystals have a different structure and electronic band structure from bulk silicon, permitting silicon nanocrystals to be applied in optical devices while bulk silicon cannot [3] and the graphene allotrope of carbon has very different quantum electronic properties from graphite [7,8] While work on silicon carbide molecular clusters has only just begun.

Using chemical intuition to explore the connectivity space in order to describe the minimum energy molecular structure of even mid-sized molecular clusters can be a labor-intensive endeavor [9]. Recent algorithmic methods have been advanced to systematize and automate quantum mechanics description of low-energy cluster isomers, for example including genetic algorithms [10], ordinary and symmetry-adapted search algorithms [11], and graph theory [12]. We developed and applied a general method for cluster connectivity analysis that combines stochastic potential surface search and pseudopotential plane-wave Density Functional Theory Car-Parinello molecular dynamics simulated annealing (PSPW-CPMD-SA) to search for stable isomeric structures.

By the results of this research we show that large silicon carbide molecular structures join delocalized carbon groups having structural similarities to structures in graphene or buckyballs with silicon groups having structural similarities to silicon nanoclusters in a myriad of combinations depending on the size of the Si_n_C_n_ molecular cluster.

## 2. Results and Discussion

Using the method outlined in the Experimental section, we studied the isomeric structures of Si_n_C_n_ clusters for values of **n** from 4 to 12. For each cluster, we determined which isomer structure had the lowest lying energy among hundreds to thousands of local minimum structures depending on the size of the Si_n_C_n_ cluster. We analyzed the structures and compared them with computed minimum energy structures found in the available literature. We report and discuss the results as follows in order of increasing **n**.

### 2.1. Si_4_C_4_

**Figure 1:**
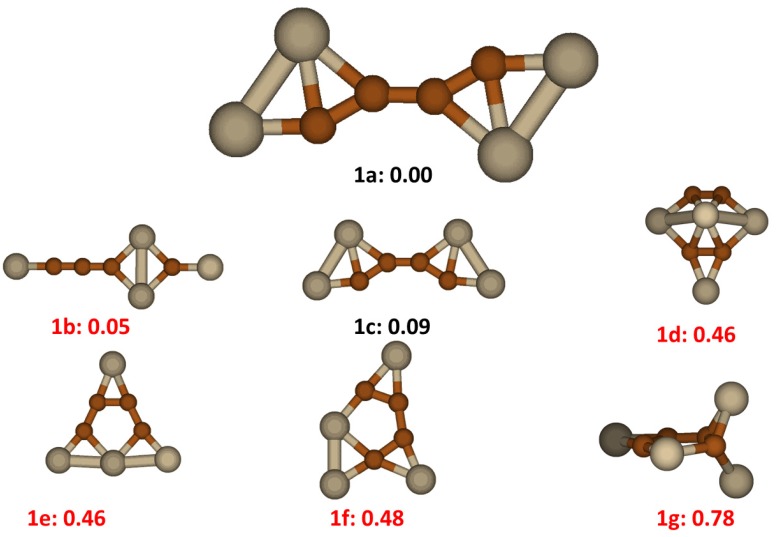
The structures and relative energies (in eV) of the lower energy Si_4_C_4_ clusters. Structures labeled in red have been previously reported in literature as **1b** [13], **1d** [14,15,16], **1e** [17], **1f** [17], **1g** [18].

The structures and relative energies of the lower energy Si_4_C_4_ clusters are displayed in Figure 1. The lowest energy structure is shown as **1a** having C_2h_ symmetry, composed of two Si_2_C_2_ groups connected by a C-C bond in *trans* positions. From another perspective view, the structure has a nearly linear SiC_4_Si conjugated bonding backbone with two Si atoms attached side by side at each end. In the backbone the average C-C bond distance is 1.34 Å and Si-C distance 1.74 Å. This *trans* isomer has a corresponding cis isomer, labeled as **1c** in Figure 1 having energy 0.09 eV higher. This isomer cluster has been studied previously by different groups [13,14,15,16,17,18] and various stable structures were reported as lowest energy structures. The low lying energy structures we found with our search method include previously published structures, labeled as **1b**, **1d**, **1f** and **1g** in Figure 1. All these previously reported structures have higher energies than **1a** ranging from 0.05 eV up to 0.78 eV. By viewing all the low lying energy structures, we see a trend that the C atoms tend to bond each other to form a conjugated segregation group while the Si atoms either cap the dangling bonds of C atom or form a dimer or trimer Si cluster.

### 2.2. Si_5_C_5_

**Figure 2:**
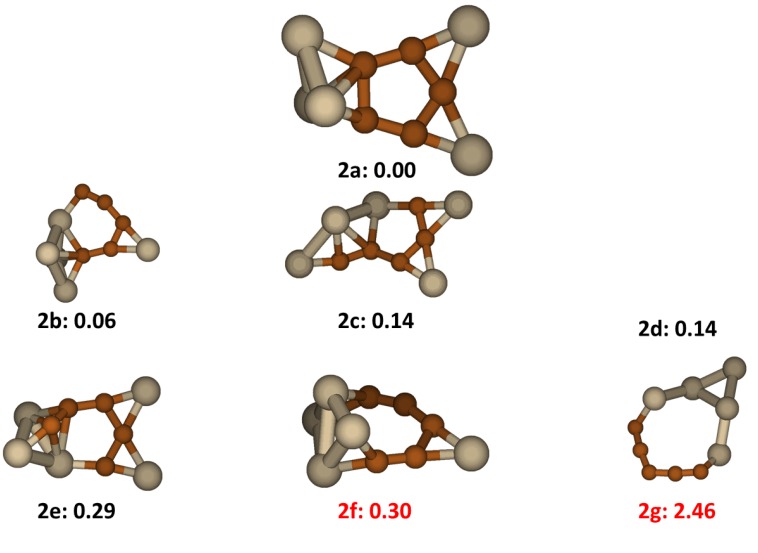
The structures and relative energies (in eV) of the lower energy Si_5_C_5_ clusters. Structure **2f** and **2g** are previously reported in ref. [17] and ref. [19], respectively.

Among Si_5_C_5_ clusters six isomer structures having the low lying energy are shown in Figure 2. The lowest energy isomer, labeled as **2a** in the figure, has a five carbon ring in the middle for which the average C-C bond length is 1.46 Å, indicating conjugation of the C_5_ group. A three-silicon triangle connects to C_5_ ring by bridging one C-C pair forming a distorted hexahedron structure. The isomer structure has Cs symmetry. All the other low energy structures involve four to six adjoining carbon atoms in a silicon-carbon ring. Structure **2b**, also having Cs symmetry, has an energy very close to that of **2a** (+0.06 eV). It has a 6-member SiC_5_ ring in the cluster. Structures **2f** and **2g** were reported previously [17,19] and they both were reproduced in this work. These two structures have significantly higher energy than the other five low lying energy structures; the isomer **2g** which has a Si_4_C_5_ 9-member ring has an especially high energy.

### 2.3. Si_6_C_6_

**Figure 3:**
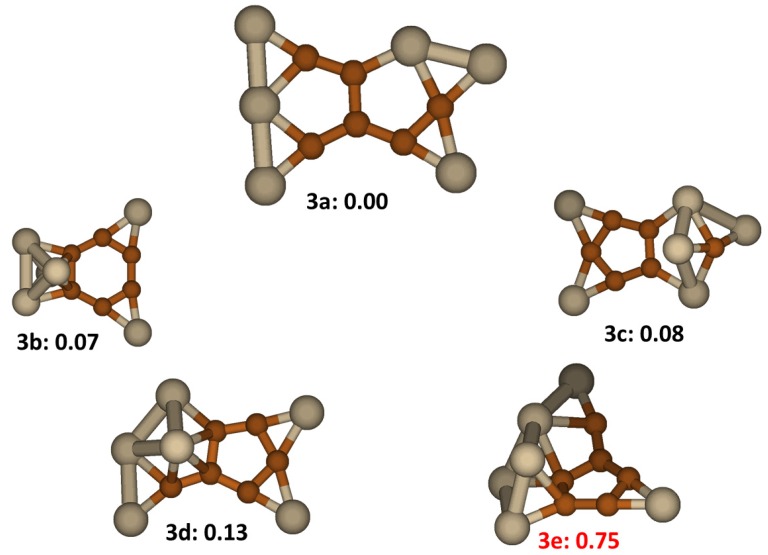
The structures and relative energies (in eV) of the lower energy Si_6_C_6_ clusters. Structure **3e** is previously reported in ref. [17].

The most stable isomer for the Si_6_C_6_ clusters was found to be a planar structure. It is discernibly more stable than the four other low energy structures shown in Figure 3. This structure, labeled as **3a**, has in its middle a pentalene type fragment with two C atoms substituted by Si atoms. In this fragment, the average C-C distance is 1.42 Å, while that of Si-C is 1.93Å, signifying a relatively strong conjugation in the C fragment and a weak Si-C bond. The whole structure is planar with Cs symmetry. Isomer **3b** is very close in energy (+0.07 eV). Its C_6_ ring in the middle has an average C-C distance of 1.43 Å also showing rather strong conjugation. The dangling bonds of C atoms are capped by 2 Si atoms and a Si_4_ cluster. All the other low lying energy structures, **3c**, **3d** and **3e**, have a C_5_ ring with an average C-C distance of 1.45 Å. Structure **3e** was reported previously as the lowest energy structure [17]. Our results show that this **3e** structure is significantly higher in energy than four other low energy isomer structures.

### 2.4. Si_7_C_7_

**Figure 4:**
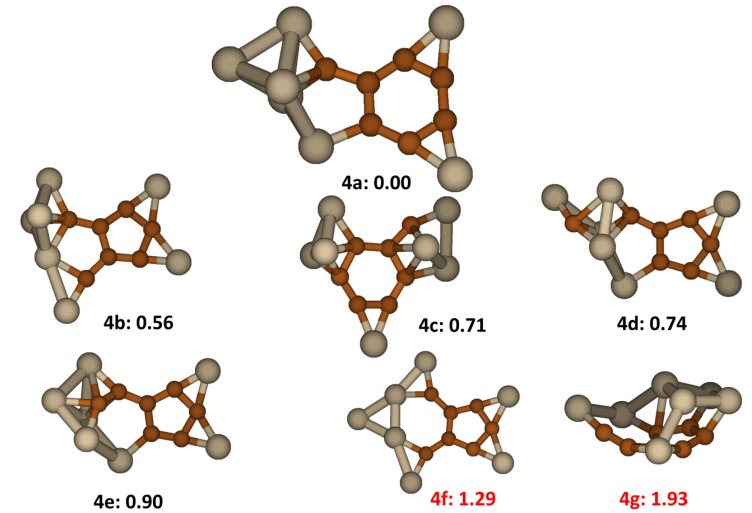
The structures and relative energies (in eV) of the lower energy Si_7_C_7_ clusters. Structure **4f** is previously reported in ref. [20] and structure **4g** is previously reported in ref. [17].

The low lying energy structures of Si_7_C_7_ clusters, displayed in Figure 4, all contain either a C_6_ ring or a C_5_ ring. The most stable isomer, **4a**, has a carbon segregation in form of a C_6_ ring with a C atom attached to it. The average C-C bond distance is 1.41 Å, which suggests conjugation in the C segregation. Dangling bonds of the carbon segregation are saturated by two individual Si atoms bridging two C-C bonds in the plane as well as by Si atoms of a Si_5_ cluster for two other carbon atoms. The whole structure has Cs symmetry. The average Si-C bond is 1.90 Å and the average Si-Si bond is 2.44 Å. The next low energy isomer, **4b**, which is significantly higher in energy compared to **4a**, has a backbone composed of C_5_ and SiC_4_ fused rings. The average C-C bond distance is 1.43 Å. Similar to **4a**, the dangling bonds of C atoms are saturated by 2 individual Si atoms and a Si_5_ cluster. The remaining low lying energy structures, except **4g**, have the similar segregation feature as **4a** or **4b**. Isomer **4f**, reported by Zang *et al*. [20] is a planar structure resembling 4b, less one Si-C linkage. It is found to have energy 1.29 eV higher than the lowest energy isomer. Isomer **4g** found in our work to have a very high energy compared to the most stable structures was also studied previously, reported in the literature as the lowest energy Si_7_C_7_ structure [17]. In **4g**, C and Si atoms form two segregation regions which then fuse to each other forming a cage like structure. This segregated cage like structure is not favored energetically compared other stable structures of Si_7_C_7_. However, this type of cage becomes more stable in clusters having higher **n**.

### 2.5. Si_8_C_8_

In the search process for the low energy isomers of the Si_8_C_8_ cluster, among tens of stable isomers we found that as in the cluster Si_7_C_7_, lower energy isomers contain carbon atom segregations of either a 5-member carbon ring (**CS5**) or a 6-member carbon ring (**CS6**) with the remainder of carbon atoms attached to the rings. By predefining **CS5** and **CS6** structures in initial geometries, we make the searching process much more efficient, avoiding a large number of high energy species. In other words, we randomly displace Si atoms around either **CS5** or **CS6** to obtain initial geometries, a concept that has analogy to parent-offspring relations in genetic search algorithms for Si clusters [10]. These predefined carbon ring structures are stable at 3000K in molecular dynamics simulations.

**Figure 5:**
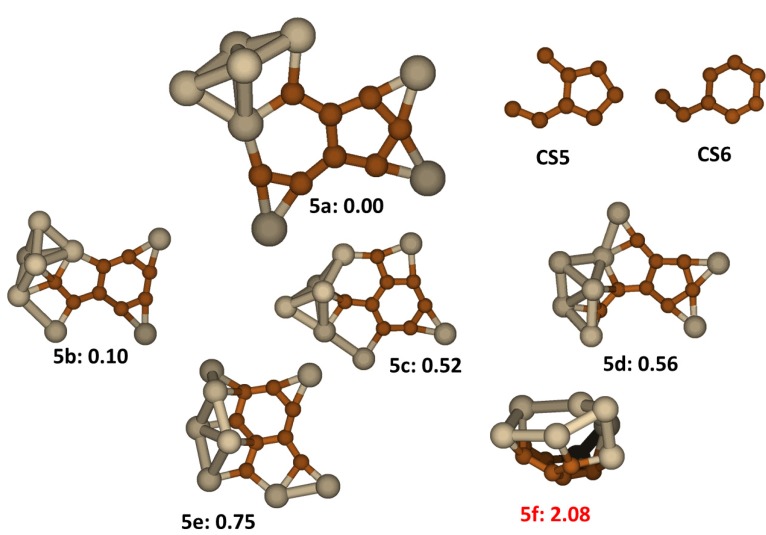
The structures and relative energies (in eV) of the lower energy Si_8_C_8_ clusters. Structure **5f** is previously reported in ref. [17].

The lowest energy structures are show in Figure 5. We find that the lowest energy isomer, **5a**, has **CS5** segregation with Cs symmetry. The dangling bonds of C atoms in C**S5** are capped in the carbon plane by three individual Si atoms and two Si atoms from a small cluster of five Si atoms. In **5a**, the average bond distance for C-C bonds is 1.43 Å, for Si-C bonds is 1.87 Å and for Si-Si bonds is 2.38 Å. Isomer **5b** which has energy +0.10 eV higher contains a **CS6** segregation and also has Cs symmetry. The rest of low-lying energy structures, except **5f**, either have **CS5** or **CS6** carbon segregation. Like **5a**, from **5b** to **5e**, all the structures have a character that a molecular plane formed by a C segregation is surrounded by individual Si atoms and a Si clusters. Structure **5f** displayed in the same figure was previously reported as the lowest energy isomer for Si_8_C_8_ [17]. In this work we could reproduce the same structure but we found it to have a much higher energy than all of the low energy structures shown in Figure 5. In structure **5f** the carbon atoms form a curved **CS5** segregation which fuses together with Si segregation forming a cage like structure similar to **4f** described above.

### 2.6. Si_9_C_9_

**Figure 6:**
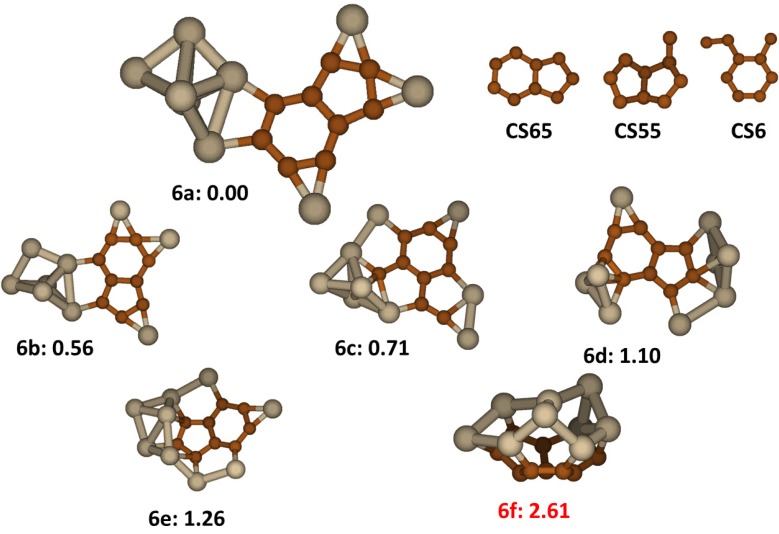
The structures and relative energies (in eV) of the lower energy Si_9_C_9_ clusters and carbon segregations. Structure **6f** is previously reported in ref. [17].

For the Si_9_C_9_ cluster there are three different stable carbon segregations identified among the tens of the lower energy isomers found in the first stage of searching. The first one is an iso-indene type of structure, *i.e.*, a fused 6-member and 5-member carbon rings (C_6_-C_5_) denoted as **CS65**, the second one is a pentalene type structure labeled as **CS55**, with two fused 5-member carbon rings (C_5_-C_5_), having one C atom attached to it; and the third one is the 6-member ring with a C atom and a C_2_ group attached in an adjacent position on the ring, denoted as **CS6**. These carbon segregations as shown in Figure 6 are all planar. As in the search process for Si_8_C_8_, after identifying these stable carbon groups, to speed up the search process we predefined these segregations to generate initial structures. The low lying energy isomers of the cluster are displayed in Figure 6 too. The most stable isomer, **6a**, has C**S65** carbon segregation and is Cs symmetric. Similar to the low lying energy isomers of Si_8_C_8_, the dangling bonds of C**S65** in the **6a** are saturated by three bridge-bonding Si atoms and a small cluster of Si_6_. The average C-C bond length in the structure is 1.42Å, the Si-C length is 1.86 Å and the Si-Si length is 2.36 Å. The next to lowest energy isomer, **6b** (+0.56 eV) has a very similar structure to **6a**, differing only by the bonding positions of the Si atoms and the small cluster. In isomer **6c**, with two Si atoms bonding **CS6** carbon segregation, the molecular plane is of form a fused 3-ring structure of C_6_-C_4_Si-C_4_Si. The rest of the low lying energy structures all contain one of the three carbon segregations. The structure **6f**, reported previously by Song *et al*. [17], unlike the other low-lying energy structures in Figure 6, is of a cage structure that is formed by a curved **CS55** segregation networked to a silicon segregation of two fused 5-member Si rings. With our finding, this structure is higher as much as 2.6 eV in energy compared to the stable non-cage structure. The structure of **6f** is actually similar to **6e** except that the Si atoms are not joined into the cage structure of **6f**, so the difference in energy between **6e** and **6f**, ~1.3 eV, represents the resolution of the stabilization energy by forming additional Si-Si bonds and the destabilizing strain energy in forming the cage, a large portion of which is associated with bending the condensed C_5_-C_6_ ring of the planar **CS55** carbon structure.

### 2.7. Si_10_C_10_

**Figure 7:**
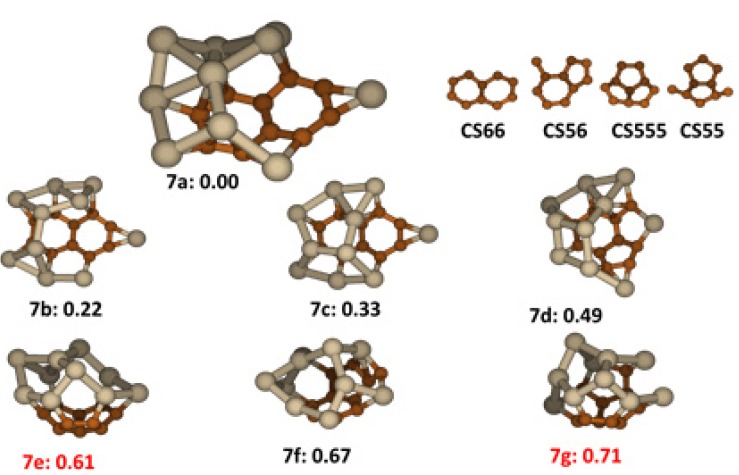
The structures and relative energies (in eV) of the lower energy Si_10_C_10_ clusters and carbon segregations. Structure **7e** and **7g** are previously reported in ref. [17] and ref. [21], respectively.

The Si_10_C_10_ cluster is sufficiently large to manifest a tendency to become a cage-like structure in low lying energy structures. Sufficient numbers of Si atoms are in the cluster to span the planar carbon segregation groups such that the strain energy is compensated by Si-Si bond formation. Four stable carbon segregations shown in Figure 7 exist among low lying energy structures of Si_10_C_10_. The first one is a naphthalene type structure labeled as **CS66**; the second one is an indene type structure labeled as C**S56**, having one C attached on the C_6_ ring; the third one is a fused three C_5_ rings denoted as C**S555** and the last one, a pentalene type structure, having two carbons attached aside of one C_5_ ring, labeled as **CS55**. The most stable structure in Figure 7, **7a**, has a **CS66** segregation networked to a segregation of nine Si atoms. A single Si atom is bridge bonded to two C atoms of **CS66** ring to cap the dangling bonds at the end of **CS66** opposite the cage attachment. The isomer is in open cage structure to maintain the most planarity of **CS66** In this structure, the average C-C bond length is 1.427Å, featuring conjugated bonding, while for Si-C and Si-Si bonds, the average lengths are 1.926 Å and 2.531 Å respectively. Strained Si-Si bonds are weaker than in smaller non-cage structures. The two isomers close in energy, **7b** and **7c**, have open-cage structures similar to **7a** differing by changes in the networks of the segregation of Si atoms. Structure **7d** has an open cage containing **CS56** carbon segregation. The rest of low lying isomers, **7e**, **7f** and **7g** are in closed cage-like structures, *i.e.*, all ten Si atoms are networked and form a cage spanning one of the carbon segregations, *i.e.*, **CS555** in structure **7e**, **CS56** in **7f** and C**S55** in **7g**. Again, by comparing the energies, we can see that an open cage structure wins the competition compromising strain energy in Si-Si bonds and in the carbon segregation. Structure **7e** and **7g** were reported as most stable structures for the cluster previously [17,21], but they both have higher energy than **7a** found in this work.

### 2.8. Si_11_C_11_

**Figure 8:**
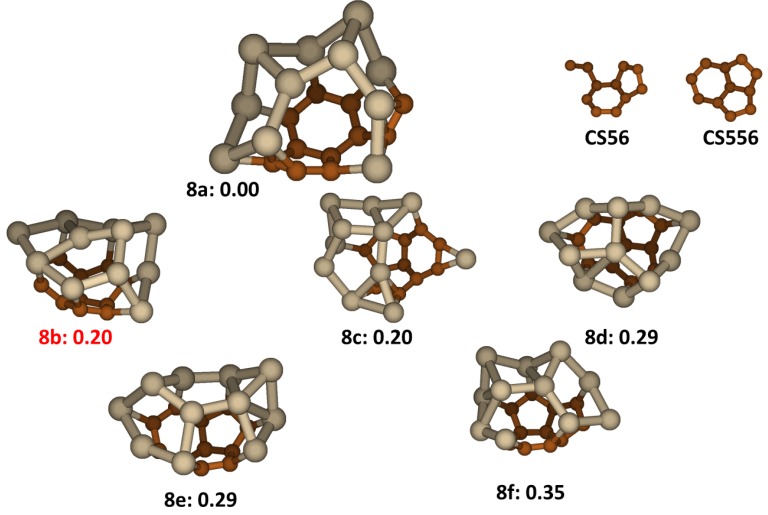
The structures and relative energies (in eV) of the lower energy Si_11_C_11_ clusters and carbon segregations. Structure **8b** is previously reported in ref. [22].

As with Si_10_C_10_, increasing numbers of total atoms in a Si_n_C_n_ cluster tends stabilize a cage structure. The Si_11_C_11_ cluster is the smallest Si_n_C_n_ cluster for which the lowest energy isomer is a fully closed cage. In Si_11_C_11_ all low lying energy structures, shown in Figure 8, have cage-like structures, among them, only one, **8c** is an open cage. There are two main carbon segregations contain in these structures as showed in Figure 8. The first one is indene type fragment with a C_2_ group attached to C_6_ ring, labeled as C**S56**. Another one is tri-cycle fragment composed of two C_5_ rings and one C_6_ ring, named as C**S556**. Both carbon segregations are curved rather than planar, permitting these structures to be spanned easier to form the cage structures. All the low lying energy isomers contain the **S556** segregation, except the lowest energy isomer, **8a**, which contains a **CS56** segregation. The larger stability of **8a** may be interpreted as that the C**S56** combines two Si atoms to make up a fused-tetra-ring structure of C_5_-C_6_-C_4_Si- C_4_Si ring. In the **8a**, the average C-C bond length is 1.428 Å while for Si-C and Si-Si bonds, the average lengths are 1.889 Å and 2.433 Å respectively. Isomer **8b** was reported earlier as the lowest structure [22], but we find that it has energy 0.20 eV higher than isomer **8a**.

### 2.9. Si_12_C_12_

**Figure 9:**
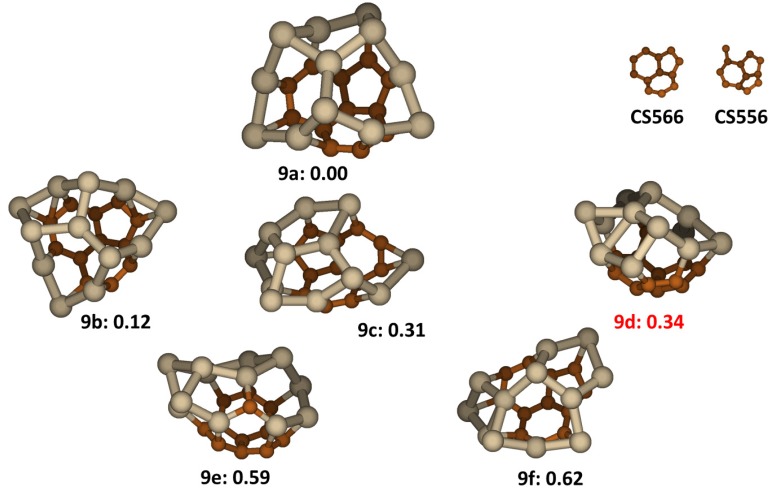
The structures and relative energies (in eV) of the lower energy Si_12_C_12_ clusters and carbon segregations. Structure **9d** is previously reported in ref. [22].

In the low-lying structures of the cluster, we identified two carbon segregations as shown in Figure 9. They both are curved and the first one (**CS665**) has two C_6_ rings and one C_5_ ring fused together while the second one (**CS655**) has one C_6_ ring and two C_5_ rings fused together. We noted that **CS665** is actually a fragment of the carbon buckyball structure consistent with its greater stability. By networking one of the carbon segregations with Si segregation, the low lying energy structures of the Si_12_C_12_ cluster tend to form closed cages as shown in Figure 9. The lowest energy structure, **9a**, has a **CS665** segregation and so do the other three low lying energy structures, **9b**, **9c** and **9f**. The average C-C, Si-C and Si-Si bond lengths in structure **9a** are 1.424 Å, 1.918 Å and 2.399 Å, respectively. The average Wiberg bond orders were 1.28 ± 0.12 for C-C bonds in the conjugated carbon segregation, 0.73 ± 0.04 for comparatively weak SiC bonds at the periphery of the carbon segregation region and 0.96 ± 0.08 for Si-Si bonds in the Si segregation region. The other two low lying structures, **9d** and **9e**, have **CS655** segregations. Structure **9d** was previously reported to be the lowest energy structure [22]. However we find that **9d** has energy about 0.34 eV higher than the lowest energy isomer, **9a**, another illustration of the advantage of our method to evaluate many randomly generated structures to find structures that have special stability.

## 3. Experimental

**Figure 10:**
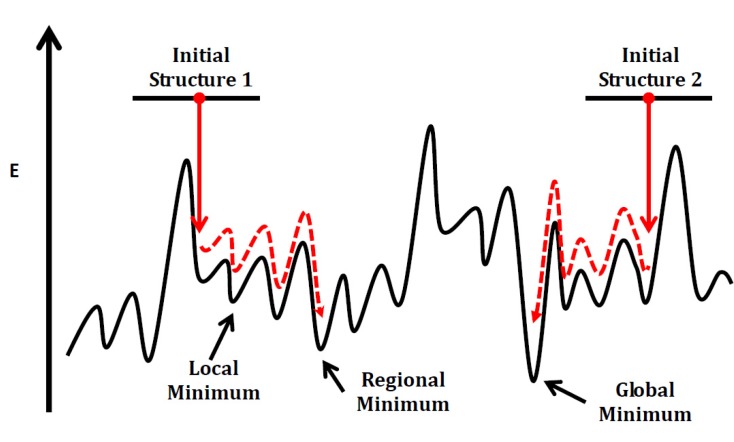
Illustrative diagram for Si_n_C_n_ cluster connecting potential surface. The red dashed lines indicate the paths of searching a regional minimum from a random-generated initial structure.

For Si_n_C_n_ clusters having equal number **n** of Si and C atoms, if **n** is greater than one there are numerous stable isomeric structures each of which is a minimum on the potential energy surface (PES). The number of stable isomers increases geometrically with **n**. As illustrated in Figure 10, if we divide a Si_n_C_n_ potential energy surface into connectivity regions that are surrounded by relatively high peaks, the minima in a region can be classified as local minima and one regional minimum. The lowest energy regional minimum should be the global minimum isomer, *i.e.*, the most stable structure for a Si_n_C_n_ cluster. Our general approach is: (a) stochastically generate a initial structure which resembles of a minimum energy structure in a potential energy connectivity region; (b) starting with the initial structure, use an efficient and reliable computational method at a moderate level of theory to search for the regional minimum structure; (c) repeat steps (a) and (b) until a sufficiently large number (hundreds to thousands) of regional minima are found; (d) choose lower energy regional minimum structures (usually 5 to10) spanning the approximate uncertainty of the moderate computational method; refine these structures at higher level of theory thereby finding a global minimum structure at the higher level of theory.

For procedure a, inspired by the stochastic search (kick) method [23], we developed a structure generator to generate at random a cluster structure. The generator program, written in FORTRAN, includes following characteristics:

(i) As in the original kick method, each atom is first placed in the origin position, and then displaced to a new position according to the three separately randomly generated distances corresponding to x, y and z coordinate axes. The maximum distance at each axis is limited to B/3, where B is the latitude of a prolate ellipsoid described below.
(ii) The new position of each atom is constrained in a sampling space with a shape of a prolate ellipsoid. The longitude of the ellipsoid is long enough to accommodate a nearly linear Si_n_C_n_ structure while the latitude is half of the longitude to include non-linear structures. If the new position of the atom is outside the sampling space, the atom is re-displaced from the current position.
(iii) In order to make the kicked structure chemically meaningful, the position of each atom in the generated structure is re-examined to know whether or not the new atom position is within an appropriate pair distance from its nearest neighboring atom(s). The selected pair distance is approximately the bond length of that pair. If the atom at the new position is not within a bonding distance of any other atom or is too close to any atom or atoms, the atom is re-displacedfrom the current position. This procedure is continued until all atoms of the structure are in a position satisfying both inclusion in the prolate ellipsoid and the bonding distance requirements.
(iv) The generator has capability to displace atoms around a predetermined fixed structural group of bonded atoms. *i.e.*, a segregation structure comparable to a functional group in organic chemistry. In many situations, normally for n ≤ 8, after analyzing regional minima of many Si_n_C_n_ isomer structures we found that some common segregation structural groups recur in many lower energy regional structures. In these cases search procedure b can be expedited by generating initial structures containing that known, fixed structure group of atoms.

To efficiently search for a regional minimum structure in procedure b, we employed the Pseudopotential Plane-Wave (PSPW) Density Functional Theory Car-Parinello Molecular Dynamics (CPMD) simulated annealing method [24,25,26,27]. It is known that PSPW approximately achieves chemical accuracy while being fast enough to rapidly model a large number of isomer systems. In combination with *ab initio* molecular dynamics (AIMD) method (e.g., Car-Parrinello), it can simulate dynamics on a ground state potential surface directly at run time. It is ideal to use PSPW-CPMD with Simulated Annealing (SA) simulations to comprehensively explore energy surface and search for low-lying isomers. The structures generated by the generator are used as the initial structure for the PSPW-CPMD-SA simulation calculations. During the annealing Nose-Hoover constant temperature is used. Initial temperatures for electrons and ions are set equal to 3,000 K. The relaxation time of 250 a.u. (2π/ω, where ω is the oscillator frequency) and 5 a.u. time step were used. An exponential cooling schedule having scaling time constant of 4.134 × 10^4^ a.u. (1ps) was used. Each structure obtained from this PSPW-CPMD-SA procedure was re-optimized at the same PSPW Density Functional Theory (DFT) level of theory. For the PSPW calculations, we used a Free-Space Boundary Condition with simple cubic super cell using a cell parameter of 20–30Å, depending on the sizes of SiC clusters. The size of the super cell selected was sufficient to avoid unphysical image interactions. A 10 hartree energy cutoff for the plane-wave wavefunctions was used. Hamann pseudopotentials were used for both Si and C atoms. Vosko’s LDA of exchange-correlation was used for PSPW DFT calculations. The PSPW-CPMD-SA simulations and PSPW-DFT re-optimizations were carried out using NWChem computational package [28]. The re-optimized structure was then archived and classified according to its total energy.

The above screening procedure from generating initial structure through PSPW-CPMD-SA, PSPW-DFT re-optimization and archiving and classifying was automated and repeated through a comprehensive shell script running in parallel on a high performance computer platform. After hundreds to thousands screening procedures, five to 10 of the lowest lying energy structures for each Si_n_C_n_ were selected for further optimization using DFT theory with full core atomic basis sets. Specifically, B3LYP hybrid functional [29] with cc-pVTZ basis sets [30] were employed using the Gaussian 09 computational chemistry package [31].

## 4. Conclusions

We designed and applied a combined approach of Stochastic Potential Surface Search and Pseudopotential Plane-Wave Car-Parinello Simulated Annealing (PSPW-CPMD-SA) to search the low-lying energy Si_n_C_n_ (n = 4–12) cluster structures. Specifically, a comprehensive random structure generator for initial Si_n_C_n_ structures was developed. Using the randomly generated initial structures as seeds, PSPW-CPMD-SA is employed as search engine to locate stable Si_n_C_n_ isomers. The iterative searching process involving initial structure generating, SA searching and energy/structure sorting is automated with an elaborated shell script. Geometry optimizations with DFT with full core atomic basis sets, specifically B3LYP/cc-pVTZ level of theory, are performed on low-lying energy isomer structures to fine tune the energy order and to determine the most stable one. The searching method was originally designed to search among Si_n_C_n_ cluster isomers, but it can be applied to search other molecular clusters, e.g., U_x_O_y_ clusters and Si_n_C_m_O clusters, as we intend to do.

By analyzing the most stable structures in isomers of Si_n_C_n_ clusters, we can see some interesting trends of how C and Si atoms bond in clusters with increasing **n**. First C atoms trend to form a conjugated segregation in the most stable isomers of a cluster. When the number of C atoms is equal to or larger than five the C segregation tends to form ring structures. These condensed ring structures are planar in smaller clusters and they will maintain planar when they grow until they yield to become curved forming more Si-C bonds with Si networked segregation spanning over it in larger clusters. For Si atoms, when clusters are small, they occur as single Si atom bridging C-C or prevalently in small tetrahedral clusters of Si_3_ rings that saturate dangling bonds at the edges of the carbon segregation. For example in Si_9_C_9_ three of the nine Si atoms separately cap five dangling bonds around the **CS65** carbon segregation while the remaining six Si atoms in form of a small cluster connect to the carbon segregation to saturate the other two carbon dangling bonds. When a Si_n_C_n_ cluster gets bigger, the Si atoms tend to form a network with each other that spans over a carbon segregation forming a cage like structure. In this way the stability gained from forming more Si-C bonds at the periphery overcomes the steric hindrance of curving a conjugated C segregation. This trend of bonding style can be seen by comparing structure **6a**, **7a** **and** **8a**. In **6a**, the most stable structure of Si_9_C_9_, carbon atoms occur in planar ring structure of **CS65** while Si atoms connect to carbon rings in single atom or small cluster. In **7a**, carbon atoms exist still in nearly planar ring structure of CS66. However, except that one Si atom caps two C atoms in C-C bond similar to the structure **6a**, the rest of nine Si atoms are networked to span over the carbon segregation making whole structure an open cage. When it comes to Si_11_C_11_, in **8a**, carbon atoms in the conjugated ring structure CS56 becomes curved in order to accept the Si_11_ network spanning over it. The structure of the minimum energy isomer **9a** for Si_12_C_12_ suggests that large Si-C clusters may be more than only theoretical interest. The intimate bonding of a carbon-segregated region that looks like conductive graphene and the bonding in the silicon-segregated region that resembles the photon-emitting structure of porous silicon suggests that these Si_12_C_12_ nanoparticles may have unusual and interesting optoelectronic properties.

In previous research describing low energy Si_n_C_m_ cluster structures, we showed that the structures of minimum energy isomers was dictated by maximizing the number of strong C-C bonds and minimizing weaker Si-Si bonds in favor of Si-C bonds. [18,32] In this research we show the consequence of this thermodynamic imperative for low-energy isomers of larger Si_n_C_n_ clusters, modified by strain effects in Si segregation and C segregation regions for cage clusters. Structures of minimum energy isomers and low energy isomers exhibit a melding of carbon segregation of condensed ring structures with silicon segregation structures by Si-C bonds. For small **n**, silicon atoms generally terminate the carbon segregation region forming 3-member Si_2_C or SiC_2_ rings. Clusters having larger **n** are characterized by formation of networks of Si_3_ and Si_4_ groups that span the carbon segregation structure.
